# Quality of Publicly Available Information About Urinary Tract Infections

**DOI:** 10.1001/jamanetworkopen.2024.44988

**Published:** 2024-11-14

**Authors:** Viktoria Schmitz, Zoe Troubh, Michael Durkin, Kevin Hsueh, Katheryn Ney, Brian D. Carpenter, Shinbee Waldron, Mary C. Politi

**Affiliations:** 1 Division of Public Health Sciences, Department of Surgery, Washington University School of Medicine in St Louis, St Louis, Missouri; 2 Division of Infectious Diseases, Department of Internal Medicine, Washington University School of Medicine in St Louis, St Louis, Missouri; 3 Department of Psychological & Brain Sciences, Washington University in St Louis, St Louis, Missouri

## Abstract

This cross-sectional study examines the content and quality of information about urinary tract infections and asymptomatic bacteriuria on publicly available websites.

## Introduction

Asymptomatic bacteriuria (ASB) is a common, benign condition in which the patient has bacteria in the urine but no symptoms of a urinary tract infection (UTI).^1^ It can be difficult to differentiate between ASB and UTIs, and antibiotic overtreatment for ASB is common, especially among older adults.^1^

Over 80% of US adults search the internet for health-related information.^2,3^ Websites with unclear information or misinformation about UTIs and ASB may influence patients’ expectations for treatment of symptoms not consistent with UTIs.^4^ Patients may also encounter inaccurate artificial intelligence (AI)–generated summaries.^5^ This cross-sectional study evaluated the content and quality of UTI and ASB information on the internet.

## Methods

Three team members (V.S., Z.T., and S.W.) performed a Google Search for UTIs in incognito mode from December 2023 to January 2024. Search terms (eAppendix 1 in Supplement 1) were identified by 4 clinicians (M.D., K.H., and 2 clinical advisory board members) specializing in geriatrics and/or infectious disease and a 5-member community advisory board of older adults and patient advocates. We reviewed the first 3 pages of results for each term, according to previous research recommendations.^6^ We reviewed websites for inclusion criteria (eg, freely accessible, English language, US-based, and designed for adult nonpregnant individuals) (eAppendix 2 and eFigure in Supplement 1).

We used a codebook to evaluate the quality of UTI diagnosis, treatment, and prevention information. The draft codebook was reviewed and revised by the study team and clinical advisors. The final codebook is shown in the eTable in Supplement 1. Websites were independently coded by 1 of 4 research coordinators (V.S., Z.T., K.N., and S.W.); questions were discussed to reach consensus. A randomly selected 20% of the websites were double coded. We followed the STROBE reporting guidelines for cross-sectional studies. Descriptive statistics (frequencies, percentages, and distribution) were performed. The institutional review board of Washington University in St Louis considered the study non–human participants research and did not require informed consent.

## Results

Of 1413 websites identified, 1082 were excluded, and 331 were included in analyses (Table 1). Almost all (322 [97%]) websites mentioned at least 1 accurate UTI symptom (Table 2). However, most (264 [80%]) included at least 1 inaccurate UTI symptom. The most commonly mentioned inaccurate symptom was change in urine color (244 [74%]). Many websites (227 [69%]) also incorrectly included strong-smelling urine as a symptom. Few websites (31 [9%]) mentioned the term *asymptomatic bacteriuria*. Even fewer (9 [3%]) mentioned that symptoms need to be present to diagnose a true UTI. A minority (70 [21%]) of websites mentioned the possibility of antibiotic resistance on an individual level. Even fewer (29 [9%]) mentioned the possibility of global antibiotic resistance, or information about adverse reactions of antibiotics (28 [8%]). A full list of symptoms, diagnosis methods, treatment, and consequences of antibiotics overuse described on websites can be found in Table 2.

**Table 1.** Characteristics of Publicly Available Websites About Urinary Tract Infections and Treatment

Type of website	Websites, No. (%) (N = 331)
Academic hospital and/or institution website	77 (23.3)
Entertainment or media website	75 (22.7)
Health care–focused organization website	72 (21.8)
General medical knowledge website	66 (19.9)
Personal website	16 (4.8)
Government website	15 (4.5)
Pharmaceutical or insurance company website	14 (4.2)
General knowledge website	6 (1.8)
Nonprofit organization website	3 (0.9)

**Table 2.** Information About UTIs and ASB on Publicly Available Websites

Type of information	Websites, No. (%) (N = 331)
Accurate symptoms	
Frequent urination or frequent urge to urinate	307 (93)
Burning while urinating	273 (82)
Pain in lower stomach and/or pelvis	269 (81)
Pain while urinating	266 (80)
Fever and/or chills	264 (80)
Blood in the urine	257 (78)
New pain in back, just below the ribs	255 (77)
New-onset memory changes or confusion	80 (24)
Leaking or new-onset incontinence	47 (14)
Newly getting up during the night to urinate	14 (4)
Inaccurate symptoms	
Change in urine color: cloudy urine, dark color urine, or light color urine	244 (74)
Strong-smelling urine or urine odor	227 (69)
Long-term and/or chronic incontinence	2 (1)
Long-term and/or chronic memory changes or confusion	2 (1)
Diagnostic methods	
Using a urine test to detect a UTI	219 (66)
Includes the term *asymptomatic bacteriuria* or *ASB*	31 (9)
Symptoms need to be present in order to diagnose true UTI	9 (3)
Using a polymerase chain reaction test to detect a UTI	4 (1)
Treatment options	
Mentions taking antibiotics	309 (93)
Mentions taking antibiotics correctly	141 (43)
Mentions taking other medications (not antibiotics) to relieve UTI symptoms such as pain	139 (42)
Mentions not taking leftover antibiotics from home	4 (1)
Consequences of antibiotics overuse when UTI symptoms are not present (ie, for ASB)	
Can lead to antibiotic resistance on a personal level	70 (21)
Can lead to antibiotic resistance at a public health level	29 (9)
Can miss other diagnoses that could be the cause of symptoms	28 (8)
Can lead to unnecessary side effects (mentions at least 1 adverse effect)	28 (8)
Can kill “good bacteria”	17 (5)

Abbreviations: ASB, asymptomatic bacteriuria; UTI, urinary tract infection.

## Discussion

The quality of information surrounding UTIs and ASB on the internet is highly variable, even among websites hosted by reputable health care–focused organizations. Although this cross-sectional study found some accurate information about UTI symptoms, diagnosis, and treatment, it was often mixed with misinformation. This misinformation can perpetuate misconceptions about UTIs and ASB, leading to inaccurate patient information and AI summaries of UTI diagnosis and management. Inaccurate information was biased toward overtreatment, which can lead to antibiotic resistance.

Study limitations include the cross-sectional design, which cannot generate statistical inference or causality. Additionally, internet-based information can change over time; included websites were rechecked before submission and were still active. We used the most common search engine, but other websites could emerge from different search engines.

Although this study evaluated UTIs, this issue could be common among other health conditions. Clinicians should work to provide accurate information and direct patients to trusted websites. Researchers could work to correct inaccurate online health information and restrict AI algorithms to trusted websites to reduce misinformation and antibiotic overuse.

## Notes

Supplement 1. **eAppendix 1. **Search Terms **eAppendix 2.** Inclusion and Exclusion Criteria **eTable.** Coding Instrument for Content of ASB/UTI Information on Websites **eFigure.** CONSORT Diagram

Supplement 2.
Data Sharing Statement
