# Supplementary material for: Quality of Publicly Available Information About Urinary Tract Infections
Source: JAMA Netw Open. 2024 Nov 14;7(11):e2444988. doi: 10.1001/jamanetworkopen.2024.44988 (PMC11565261; doi:10.1001/jamanetworkopen.2024.44988)
Supplement: Supplement 1. — eAppendix 1. Search Terms eAppendix 2. Inclusion and Exclusion Criteria eTable. Coding Instrument for Content of ASB/UTI Information on Websites eFigure. CONSORT Diagram [file jamanetwopen-e2444988-s001.pdf]

Supplemental Online Content

Schmitz V, Troubh Z, Durkin M, et al. The quality of publicly available information about urinary tract infections. *JAMA Netw Open*. 2024;7(11):e2444988.
doi:10.1001/jamanetworkopen.2024.44988

eAppendix 1. Search Terms

eAppendix 2. Inclusion and Exclusion Criteria

eTable. Coding Instrument for Content of ASB/UTI Information on Websites

eFigure. CONSORT Diagram

This supplemental material has been provided by the authors to give readers additional information about their work.

© 2024 Schmitz V et al. *JAMA Network Open*.

eAppendix 1. Search Terms

"UTI," "urinary tract infection," "kidney infection," "bladder infection," "asymptomatic bacteriuria," "antibiotics UTI," "symptoms of a bladder infection," "when to treat a UTI," "antibiotics kidney infection," "antibiotics bladder infection," "symptoms of UTI," and "symptoms of kidney infection."

eAppendix 2: Inclusion and Exclusion Criteria

Websites were included if they contained information about UTIs, were publicly available, were designed for adult non-pregnant patients and were based in the United States. Websites were excluded if they were a duplicate, paid advertisement, contained no information about UTIs, were based outside of the United States (because of different guidelines and recommendations internationally), only contained short dictionary definitions without additional information, required a login, or were designed for medical professionals. Websites were also excluded if they were specifically designed for children, pregnant women, individuals with specific medical conditions (e.g. prior to surgery, Autosomal Dominant Polycystic Kidney Disease, catheter-associated UTIs), atypical presentation in immunocompromised patients, or referred to conditions in non-humans (i.e., animals).

© 2024 Schmitz V et al. *JAMA Network Open*.

eTable. Coding Instrument for Content of ASB/UTI Information on Websites

Website name:	
Website url:	
Additional urls linked to:	
Date of data collection:	
Research team member:	
1. Does the website contain information about UTIs (general description about what they are, their diagnosis, and their treatment; exclude websites that mention it as a list of common concerns but with no details about them)?	Yes
	No
2. Is the website publicly available with no login information required?	Yes
	No
3. Is the website for patients and the public (rather than only for clinicians, clinical journals)?	Yes
	No
4. Is the website for adults (not only for children or pregnant women)?	Yes
	No
4a. Is the website for older adults specifically? (Optional, not necessary to continue)	Yes
	No
5. Is the website US-based (not international)?	Yes
	No

If the website meets the above inclusion criteria, continue below.

© 2024 Schmitz V et al. *JAMA Network Open*.

eTable cont

	Mentioned Y/N
**Content of the website**	
**1.** Overview of UTIs and their treatment	
**1.A** Does the website describe what urinary tract infections are? Example: A common bacterial infection of any part of the urinary system, presenting with urinary symptoms, and can be treated with antibiotics.	Yes
	No
**1.B** Does the website use other terms for urinary tract infection or its progression (ex: kidney infection, bladder infection, cystitis)?	Yes
	No
**1.C** Does the website contain information about possible spread of infection if left untreated, or potential hospitalization from UTIs?	Yes
	No
**1.D** Does it describe how UTIs are treated?	Yes
	No
**2.** Does the website list the following accurate risk factors for getting a urinary tract infection?	
**2.A** Sexual intercourse	Yes
	No
**2.B** Pregnancy (often treated even if asymptomatic)	Yes
	No
**2.C** Age	Yes
	No
**2.D** Female anatomy	Yes
	No
**2.E** Compromised immune system	Yes
	No
**2.F** Catheter	Yes
	No
**2.G** Urinary procedure	Yes
	No
**2.H** Menopause	Yes
	No
**2. I** Urinary tract blockages (e.g. kidney stones)	Yes

© 2024 Schmitz V et al. *JAMA Network Open*.

	No
**2. J** Diabetes	Yes
	No
**3.** Does the website list the following inaccurate or unclear risk factors as accurate risk factors for getting a urinary tract infection?	
**3.A** Poor hygiene (other than related to female anatomy, e.g. not changing underwear, not washing hands, using the same toilet as someone else)	Yes
	No
**3.B** Holding in urine	Yes
	No
**3.C** Only women can get UTIs	Yes
	No
**3.D** UTIs are contagious (includes classifying as an STD)	Yes
	No
**3.E** Swimming or taking a bath	Yes
	No
**3.F** Specific foods or drinks (including acidic foods)	Yes
	No
**4.** Diagnosis	
**4.A** Does the website mention using a urine test to detect a urinary tract infection (urinalysis, reflex culture, dip stick)?	Yes
	No
**4.B** Does the website mention using a PCR-based test to detect a urinary tract infection?	Yes
	No
**4.C** Does the website mention that symptoms need to be present to diagnose a true urinary tract infection?	Yes
	No
**4.D** Does the website mention the term "asymptomatic bacteriuria" or ASB (bacteria in the urine with no symptoms)?	Yes
	No
**5.** Does the website describe accurate UTI Symptoms?	
**5.A** Burning while urinating	Yes
	No
**5.B** Pain while urinating (dysuria)	Yes
	No

© 2024 Schmitz V et al. *JAMA Network Open*.

**5.C** Frequent urination or a frequent urge to urinate	Yes
	No
**5.D** Leaking or new onset incontinence	Yes
	No
**5.E** Newly getting up during the night to urinate (night waking)	Yes
	No
**5.F** New onset memory changes or confusion	Yes
	No
**5.G** Blood in the urine (hematuria)	Yes
	No
**5.H** Pain in lower stomach/pelvis	Yes
	No
**5.I** Fever and/or chills	Yes
	No
**5.J** New pain in back, just below the ribs	Yes
	No
**5.K** What UTI symptom was mentioned first?<br>a) Burning while urinating<br>b) Pain while urinating (dysuria)<br>c) Frequent urination or a frequent urge to urinate<br>d) Leaking or new onset incontinence<br>e) Newly getting up during the night to urinate (night waking)<br>f) New onset memory changes or confusion<br>g) Blood in the urine (hematuria)<br>h) Pain in lower stomach/pelvis<br>i) Fever and/or chills<br>j) New pain in back, just below the ribs<br>k) Cloudy (NON SYMPTOMS)<br>l) Foul or strong odor (NON SYMPTOMS)<br>m) Diarrhea<br>n) Puss<br>o) nausea<br>p) None	
**6.** Does the website mention other <u>inaccurate or unclear symptoms</u> as true UTI symptoms?	
**6.A** Strong smelling urine	Yes
	No

© 2024 Schmitz V et al. *JAMA Network Open*.

**6.B** Change in urine color: Cloudy urine, dark color urine or light color urine	Yes
	No
**6.C** Long-term/chronic memory changes or confusion	Yes
	No
**6.D** Long-term/chronic incontinence	Yes
	No
**6.E** Other (fill in__________________)	
**7.** Does the website mention treatment for UTIs?	
**7.A** Mentions taking antibiotics	Yes
	No
**7.B** Mentions taking other medications (not antibiotics) to treat urinary tract infections to relieve symptoms	Yes
	No
**7.C** Mentions taking antibiotics correctly (ex: taking the full prescription)	Yes
	No
**7.D** Mentions not taking leftover antibiotics from home	Yes
	No
**8.** Does the website mention consequences for antibiotic overtreatment of ASB or in general?	
**8.A** Can kill "good bacteria"	Yes
	No
**8.B** Can lead to antibiotic resistance on a personal level	Yes
	No
**8.C** Can lead to antibiotic resistance at a public health level	Yes
	No
**8.D** Can miss other diagnoses that could be the cause of symptoms	Yes
	No
**9.** Does the website mention side effects of antibiotic treatment?	
**9.A** Rash	Yes
	No
**9.B** Diarrhea	Yes
	No

© 2024 Schmitz V et al. *JAMA Network Open*.

**9.C** Throwing up	Yes
	No
**9.D** Headaches	Yes
	No
**9.E** Tendon damage	Yes
	No
**9.F** Nerve damage	Yes
	No
**9.G** Yeast infection	Yes
	No
**10.** Does the website mention <u>ways to prevent UTIs</u> for infrequent UTIs?	
**10.A** Drinking water/staying hydrated	Yes
	No
**10.B** Emptying bladder after intercourse	Yes
	No
**10.C** Women should wipe front to back	Yes
	No
**11.** Does the website mention <u>inaccurate or unclear prevention</u> strategies for infrequent UTIs?	
**11.A** Taking cranberry supplements or cranberry juice (other than for recurring UTIs)	Yes
	No
**11.B** Taking antibiotics (other than for recurring UTIs)	Yes
	No
**11.C** Vaginal estrogen (other than for recurring UTIs)	Yes
	No
**12.** Other characteristics of the website	
**12.A** The website provides the author names	Yes
	No
**12.B** The website provides the author credentials	Yes
	No
**12.C** The website provides publication date	Yes
	No
**12.D** The website discloses the funding site	Yes
	No
**12.E** The website provides citations for evidence	Yes

© 2024 Schmitz V et al. *JAMA Network Open*.

	No
**12.F** The website provides the date of last update	Yes
	No
**13.** Type of organization that hosts the site	
**13.A** Health care-focused organization (e.g. Choosing Wisely, American Geriatrics Society, Urology Care Foundation)	Yes
	No
**13.B** Hospital/academic medical center or academic institution (e.g. Mayo Clinic, Washington University, Cleveland Clinic)	Yes
	No
**13.C** Non-profit organization (e.g. AARP)	Yes
	No
**13.D** General knowledge website (e.g. Wikipedia)	Yes
	No
**13.E** General medical knowledge website (e.g. WebMD, GoodRx)	Yes
	No
**13.F** Pharmaceutical or insurance company (e.g. Pfizer, Eli Lily, United, Aetna, etc.)	Yes
	No
**13.G** Entertainment or media site (e.g. Health magazine)	Yes
	No
**13.H** Government website (e.g. CDC, FDA)	Yes
	No
**13.I** Personal website (not a blog, e.g. Dr. website)	Yes
	No
**13.J** Other (Please describe)	
**14.** If risks/benefits were stated: How were they numerically quantified?	
**14.A** Percent	Yes
	No
**14.B** Frequencies or absolute numbers	Yes
	No
**14.C** Other format (e.g., 1 in X, relative risk “twice as likely to…”)	Yes
	No

© 2024 Schmitz V et al. *JAMA Network Open*.

**14.D** Inconsistent/contradictory information about UTIs within the site	Yes
	No
**14.E** If yes, describe:	
**14.F** Is the website available in another language (i.e. not through manual translation)?	Yes
	No
**14.G** Does the website have a printable section for a patient to save (i.e. to share with family or clinicians)?	Yes
	No
**NOTES:**	

© 2024 Schmitz V et al. *JAMA Network Open*.

eFigure: CONSORT Diagram

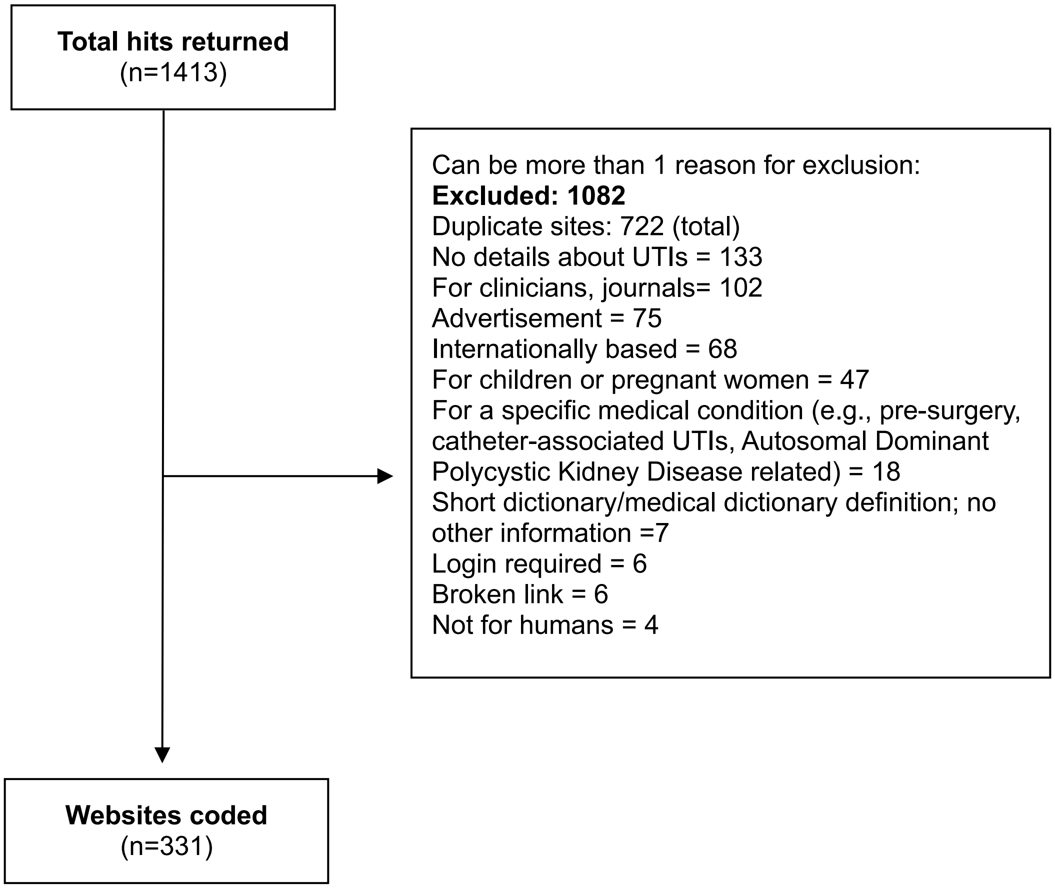

© 2024 Schmitz V et al. *JAMA Network Open*.
