# Supplementary material for: Quality of Publicly Available Information About Urinary Tract Infections
Source: JAMA Netw Open. 2024 Nov 14;7(11):e2444988. doi: 10.1001/jamanetworkopen.2024.44988 (PMC11565261; doi:10.1001/jamanetworkopen.2024.44988)
Supplement: Supplement 2. — Data Sharing Statement [file jamanetwopen-e2444988-s002.pdf]

Data Sharing Statement

Schmitz. The Quality of Publicly Available Information About Urinary Tract Infections. *JAMA Netw Open*. Published November 14, 2024. doi:10.1001/jamanetworkopen.2024.44988

Data
Data available: Yes
Data types: Data (not involving human participants)
How to access data: vschmitz@wustl.edu
When available: With publication

Supporting Documents
Document types: None

Additional Information
Who can access the data: Anyone requesting the data
Types of analyses: For a stated purpose
Mechanisms of data availability: After approval of a brief proposal indicating use
